# Clinical benefit of long-term lipoprotein apheresis in patients with severe hypercholesterolemia or Lp(a)-hyperlipoproteinemia with progressive cardiovascular disease

**DOI:** 10.1007/s11789-015-0071-3

**Published:** 2015-02-12

**Authors:** Franz Heigl, Reinhard Hettich, Norbert Lotz, Harduin Reeg, Tobias Pflederer, Dirk Osterkorn, Klaus Osterkorn, Reinhard Klingel

**Affiliations:** 1 Dres. Heigl, Hettich, and Partner, Medical Care Center Kempten-Allgaeu, Robert-Weixler-Straße 19, 87439 Kempten, Germany; 2 Medical Economics Institute, Zieblandstraße 9, 80799 Munich, Germany; 3 Apheresis Research Institute, Stadtwaldgürtel 77, 50935 Cologne, Germany

**Keywords:** LDL cholesterol, Lipoprotein(a), Cardiovascular events, Lipoprotein apheresis, LDL-Cholesterin, Lipoprotein(a), Kardiovaskuläre Ereignisse, Lipoproteinapherese

## Abstract

Low-density lipoprotein cholesterol (LDL-C) and lipoprotein(a) (Lp(a)) are established causal risk factors for cardiovascular disease (CVD). Efficacy, safety, and tolerability of lipoprotein apheresis (LA) were investigated in 118 patients with CVD covering a period with 36,745 LA treatments in a retrospective, monocentric study. Indications for LA were severe hypercholesterolemia (*n* = 83) or isolated Lp(a) hyperlipoproteinemia (Lp(a)-HLP) (*n* = 35). In patients with hypercholesterolemia, initial pre-LA LDL-C was 176.4 ± 67.0 mg/dL. In patients with isolated Lp(a)-HLP, initial pre-LA Lp(a) was 127.2 ± 67.3 mg/dL. Mean reduction rates of LA were 67 % for both LDL-C and Lp(a). During chronic LA, the average annual rate of major adverse cardiac events of all patients declined by 79.7 % (*p* < 0.0001). Subgroup analysis showed decline by 73.7 % (*p* < 0.0001) in patients with severe hypercholesterolemia, and by 90.4 % (*p* < 0.0001) in patients with isolated Lp(a)-HLP. Adverse events occurred in 1.1 % of treatments. LA treatment of patients with a high risk for CVD due to hypercholesterolemia and/or Lp(a)-HLP demonstrated clinical benefit and was safe and well tolerated.

## Abstract (de)

LDL-Cholesterin (LDL-C) und Lipoprotein(a) (Lp(a)) sind etablierte Risikofaktoren für kardiovaskuläre Erkrankungen (CVD). Wirksamkeit, Sicherheit und Verträglichkeit der Lipoproteinapherese (LA) wurden bei 118 Patienten mit CVD im Rahmen einer retrospektiven monozentrischen Studie untersucht, in der 36.745 LA-Behandlungen zur Auswertung kamen. LA-Indikationen waren schwere Hypercholesterinämie (*n* = 83) oder isolierte Lp(a)-Hyperlipoproteinämie (Lp(a)-HLP) (*n* = 35). Bei den Patienten mit Hypercholesterinämie lag der initiale LDL-Cholesterinspiegel vor Einleitung der Apheresebehandlung bei 176,4 ± 67,0 mg/dL. Bei den Patienten mit isolierter Lp(a)-HLP betrug der ursprüngliche Lp(a)-Spiegel 127,2 ± 67,3 mg/dL. Die mittleren Reduktionsraten, die durch die LA erreicht werden konnten, lagen sowohl für LDL-C als auch für Lp(a) bei 67 %. Während der chronischen LA-Behandlung fiel die mittlere jährliche Rate schwerwiegender kardialer Ereignisse (MACE) im Gesamt-Patientenkollektiv um 79,7 % (*p* < 0,0001). Eine Subgruppenanalyse zeigte bei Patienten mit schwerer Hypercholesterinämie eine MACE-Reduktion um 73,7 % (*p* < 0,0001) und bei den Patienten mit isolierter Lp(a)-HLP um 90,4 % (*p* < 0,001). Unerwünschte Ereignisse (AE) traten bei 1,1 % der Behandlungen auf. Insgesamt erwies sich die LA-Behandlung von Patienten mit hohem CVD-Risiko aufgrund einer schweren Hypercholesterinämie und/oder Lp(a)-HLP als klinisch wirksam, sicher und gut verträglich.

## Introduction

LDL-C has been recognized as most important risk factor for coronary artery disease (CAD) for more than 30 years [1]. In particular, statin trials established a clear link between therapeutic lowering of low-density lipoprotein cholesterol (LDL-C) and reduced incidence of cardiovascular event rates [2, 3]. In recent years, the equally atherogenic, thrombogenic, and inflammatory potential of lipoprotein(a) (Lp(a)), which was first identified by K. Berg in 1963, has gained increasing attention [4–9]. After withdrawal of nicotinic acid in Europe in January 2013, there is no pharmacological treatment available to lower an elevated Lp(a) level significantly. Unlike with hypercholesterolemia, it was unclear for a long time whether Lp(a) level reduction would improve cardiovascular outcome.

Lipoprotein apheresis (LA) treatment can effectively lower LDL-C as well as Lp(a) by 60–80 % during a single treatment session. After encouraging experiences in individual patients with isolated Lp(a) hyperlipoproteinemia (Lp(a)-HLP), results of LA treatment were published for this new indication in a multicenter, longitudinal cohort study with 120 patients [10]. Reduction of the Lp(a) level by LA treatment resulted in a decline of the per-year and per-patient major adverse cardiac event (MACE) count from 1.06 to 0.14, representing a reduction of 86 %. Due to methodological weaknesses in this study and considering costs of LA reimbursement, a prospective study was stipulated by German authorities. A randomized design of the study, which was initially suggested, was rejected by ethics committees in view of the favorable results of the retrospective study. In the multicenter study “Pro(a)LiFe,” 170 patients were included after approval for chronic LA due to isolated Lp(a)-HLP according to the German reimbursement authority Federal Joint Committee (GBA) [11]. Observation periods of 2 years before and 2 years after commencing LA treatment demonstrated decline of the annual per-patient MACE rate from 0.41 ± 0.45 to 0.09 ± 0.22 meaning a significant reduction of 78 %. Overall, this prospective study fully confirmed results of the earlier retrospective study.

A monocentric, retrospective, longitudinal cohort study was conducted at our medical competence center for apheresis, performing nearly 6000 LA treatments per year. All investigated patients had been approved for chronic LA treatment according to the guidelines of GBA, following an initial and annually renewable application, due to the following diagnoses: severe hypercholesterolemia or isolated Lp(a)-HLP with progressive CVD [12]. Complete details of this study have been published elsewhere [13].

## Results

### Patient characteristics

The study included 118 consecutive patients who received chronic LA treatment between October 1996 and December 2013 at our center for a mean individual period of 6.8 ± 4.9 (range, 1–23) years. This amounted to a total of 797 treatment years including 36,745 single treatment sessions. Patient characteristics are summarized in Table 1. Indication for LA treatment included severe hypercholesterolemia in approximately 70 % and isolated Lp(a)-HLP of > 60 mg/dL with progressive CVD in approximately 30 %. To fulfill the reimbursement criteria of isolated Lp(a)-HLP, LDL-C levels were treated by maximally tolerated lipid-lowering medication to reach the target level of < 100 mg/dL as part of optimized control of all other cardiovascular risk factors. Irrespective of the criteria for LA indication, there was a substantial overlap between the two dyslipidemia types in the 118 patients: 111 had hypercholesterolemia requiring lipid-lowering medication (94.1 %) and 83 patients had Lp(a)-HLP (70.3 %).

**Table 1:** Baseline characteristics at the time of first lipoprotein apheresis treatment

	All patients (*n* = 118)
Male/female	77 (65.3 %)/41 (34.7 %)
Age, years	59.6 ± 11.2 (range, 25–83)
Male, years	58.1 ± 10.5 (range, 37–81)
Female, years	62.5 ± 11.9 (range, 25–83)
Body mass index, kg/m^2^	27.7 ± 4.2
Cardiovascular risk factors	
Positive family history	79 (67.0 %)
Hypercholesterolemia	111(94.1 %)
Lp(a) hyperlipoproteinemia	83 (70.3 %)
Arterial hypertension	72 (61.0 %)
Diabetes mellitus	28 (23.7 %)
Smoking habits	
Never	69 (58.5 %)
Former	47 (39.8 %)
Current	2 (1.7 %)
Chronic renal failure as assessed by Cockcroft–Gault formula: eGFR, mL/min	
30–59	18 (15.3 %)
15–29	2 (1.7 %)
< 15 or dialysis	7 (5.9 %)
Cardiovascular diseases	
Coronary artery disease (CAD)	118 (100 %)
One-vessel CAD	16 (13.5 %)
Two-vessel CAD	18 (15.3 %)
Three-vessel CAD	84 (71.2 %)
Cerebrovascular disease	94 (79.7 %)
Peripheral artery disease	27 (22.9 %)
Indication for LA	
Severe hypercholesterolemia	83 (70.3 %)
Treatment failure	10 (12.0 %)
Intolerability of medical treatment	73 (88.0 %)
Isolated Lp(a) elevation	35 (29.7 %)
Time between first cardiovascular event and first LA, years	6.4 ± 5.6 (range, 1–27)

79.3 % of nonhemodialysis patients were treated via peripheral venous access, whereas 20.7 % required an AV shunt. The average interval between treatments was 1 week resulting in a mean of 51.3 treatments per patient per year. Use of different LA methods and treatment volumes of plasma and blood are summarized in Table 2.

**Table 2:** Lipoprotein apheresis (LA) methods and treated plasma and blood volumes

LA method	Number of treatments (*n* = 36,745)	Plasma (P) and blood (B) volumes, mL per treatment
Heparin-induced extracorporeal LDL precipitation apheresis	17,758 (48.3 %)	3103 ± 705 (P)
Temperature optimized Double filtration plasmapheresis	9370 (25.5 %)	3100 ± 675 (P)
Polyacrylate adsorption (Direct adsorption of lipoproteins from whole blood)	9218 (25.1 %)	8129 ± 1,173 (B)
Simple DFPP (Membrane filtration optimised novel extracorporeal treatment)	399 (1.1 %)	3600 ± 842 (P)

### Medication and laboratory parameters

All patients were receiving maximally tolerated lipid-lowering medication and individually optimized cardiac medication prior to and during each stage of LA treatment. Notably, these included platelet aggregation inhibitors, beta blockers, ACE inhibitors, and angiotensin receptor blockers as well as diuretics. The contraindication of ACE inhibitors with extracorporeal adsorption techniques had to be considered.

Laboratory parameters before and during chronic LA treatments are summarized in Tables 3 and 4. The mean interval value C_mean_ between two LA treatments was calculated according to the formula suggested by Kroon [14]: C_interval mean_ = C_min_ + 0.73 (C_max_ − C_min_), where C_min_ corresponds to the minimal LDL-C or Lp(a) concentrations right after LA treatment and C_max_ expresses the LDL-C or Lp(a) concentrations immediately before the next LA treatment [14]. Patients with the indication of severe hypercholesterolemia also exhibited elevated Lp(a) levels with an average of 75.0 mg/dL in 57.8 % (48 of 83) patients. The upper average limit could be lowered simultaneously with that of LDL-C, with equal efficacy per session but by 56.4 % regarding average levels of long-term treatment. So the long-term Lp(a) reduction was substantially higher compared with LDL-C reduction by only 32.1 %. In the patient group with isolated Lp(a) elevation, average levels of Lp(a) could be reduced by 52,8 %, whereas LDL-C was only lowered by 22,7 % (Table 4).

**Table 3:** Plasma concentrations of low-density lipoprotein cholesterol (LDL-C) and lipoprotein(a) (Lp(a)) in patients with severe hypercholesterolemia before, in first month, and during steady state of chronic lipoprotein apheresis (LA)

*LDL-C*	*Before LA*	*First month*	*Steady state*	*Mean total LDL-C reduction (%)*
C_max_ before LA (mg/dL)	Not done	154.7 ± 51.4	148.8 ± 43.9	
C_min_ after LA (mg/dL)	Not done	63.1 ± 26.8	48.9 ± 18.6	
Reduction by LA (%)	Not done	58.7 ± 12.0	66.7 ± 10.8 (*p <* 0.0001)	
Interval mean level (mg/dL)	176.4 ± 67.0	128.1 ± 42.6	119.8 ± 34.7	32.1 ± 19.6 (*p <* 0.0001)
*Lp(a)*	*Before LA*	*First month*	*Steady state*	*Mean total Lp(a) reduction (%)*
C_max_ before LA (mg/dL)	Not done	52.5 ± 55.4	40.2 ± 38.9	
C_min_ after LA (mg/dL)	Not done	23.4 ± 25.2	14.0 ± 12.6	
Reduction by LA (%)	Not done	54.6 ± 20.7	65.2 ± 30.7 (*p <* 0.0001)	
Interval mean level (mg/dL)	75.0 ± 64.5	48.3 ± 47.2	32.7 ± 31.0	56.4 ± 20.5 (*p <* 0.0001)

There have been patients in whom no Lp(a) was detectable, but they have not been separately recorded

**Table 4:** Plasma concentrations of lipoprotein(a) (Lp(a)) and low-density lipoprotein cholesterol (LDL-C) in patients with isolated Lp(a) elevation before, in first month, and during steady state of chronic lipoprotein apheresis (LA)

*Lp(a)*	*Before LA*	*First month*	*Steady state*	*Mean total Lp(a) reduction (%)*
C_max_ before LA (mg/dL)	Not done	97.2 ± 56.0	74.5 ± 24.3	
C_min_ after LA (mg/dL)	Not done	39.6 ± 23.1	24.5 ± 8.9	
Reduction by LA (%)	Not done	59.5 ± 11.9	66.8 ± 5.8 (*p <* 0.0001)	
Interval mean level (mg/dL)	127.2 ± 67.3	81.2 ± 45.8	60.0 ± 19.5	52.8 ± 23.0 (*p <* 0.0001)
*LDL-C*	*Before LA*	*First month*	*Steady state*	*Mean total LDL-C reduction (%)*
C_max_ before LA (mg/dL)	Not done	92.6 ± 33.9	91.7 ± 27.9	
C_min_ after LA (mg/dL)	Not done	41.0 ± 18.2	31.8 ± 15.6	
Reduction by LA (%)	Not done	55.3 ± 12.9	65.7 ± 8.7 (*p <* 0.0001)	
Interval mean level (mg/dL)	96.1 ± 33.5	77.7 ± 28.1	74.3 ± 23.7	22.7 ± 31.2 (*p <* 0.0001)

### Analysis of events

Analysis of events was performed for 118 patients who were observed for an average of 6.4 ± 5.6 (range, 1–27) years between their first cardiovascular event and the beginning of LA treatment and were then observed for an average of 6.8 ± 4.9 (range, 1–23) years during chronic LA treatment. In accord with other authors’ definitions [10, 11], cardiac death, a non-lethal myocardial infarction (MI), coronary bypass surgery (CABG), and percutaneous coronary intervention or stent implantation (PCI) all counted as MACEs representing the primary composite outcome parameter. Adverse cardiac and vascular events (ACVEs) made up the secondary composite outcome parameter, meaning all coronary as well as vascular events in all noncardiac vascular regions. The absolute number of cardiovascular events (MACEs and ACVEs) preceding and following initiation of chronic LA treatment and derived reduction rates for all investigated patients and for the patients of both treatment indications are shown in Table 5. During this period, the average annual MACE rate per patient could be reduced from 0.35 to 0.07 in the total of 118 patients. The average reduction rate after initiation of LA treatment was 79.7 % for MACEs and 73.3 % for ACVEs.

**Table 5:** Total number of major adverse coronary events (MACEs) and adverse cardiac and vascular events (ACVEs) in 6.4 ± 5.6 (range, 1–27) years before and 6.8 ± 4.9 (range, 1–23) years after commencing chronic lipoprotein apheresis (LA)

	*All patients (n = 118)*		
	Before LA (*n*)	During LA (*n*)	Reduction rate (%)
MACE	261	53	79.7 (*p* < 0.0001)
Myocardial infarction	68	10	85.3 (*p* < 0.0001)
Percutaneous coronary intervention	138	37	73.2 (*p* < 0.0001)
Coronary artery bypass graft	55	6	89.1 (*p* < 0.0001)
ACVE	289	77	73.3 (*p* < 0.0001)
*Patients with severe hypercholesterolemia (n = 83)*			
MACE	167	44	73.7 (*p* < 0.0001)
Myocardial infarction	48	8	83.3 (*p* < 0.0001)
Percutaneous coronary intervention	81	31	61.7 (*p* < 0.0001)
Coronary artery bypass graft	38	5	86.8 (*p* < 0.0001)
ACVE	184	66	64.1 (*p* < 0.0001)
*Patients with isolated Lp(a) elevation (n = 35)*			
MACE	94	9	90.4 (*p* < 0.0001)
Myocardial infarction	20	2	90.0 (*p* < 0.0001)
Percutaneous coronary intervention	57	6	89.5 (*p* < 0.0001)
Coronary artery bypass graft	17	1	94.1 (*p* < 0.0001)
ACVE	105	11	89.5 (*p* < 0.0001)

**Fig. 1:**
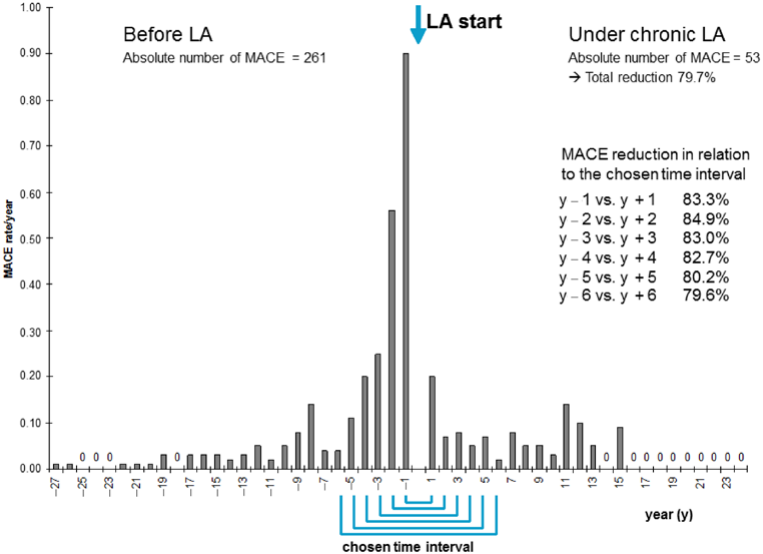
Major adverse coronary event (MACE) rates per year in relation to the number of observed patients and in relation to the chosen time interval. All patients (*n* = 118) with severe hypercholesterolemia (*n* = 83) and isolated lipoprotein(a) (Lp(a)) elevation (*n* = 35) are included in this figure (LA lipoprotein apheresis)

Figure 1 shows the number of annual MACE rates with related numbers of patients before and during LA treatment, i.e., MACE rate per patient per year. The number of analyzed patients varied in years during LA treatment due to the retrospective design. The MACE rate increased continuously prior to LA treatment from 0.04 in year − 6 to 0.9 in year − 1. Once LA treatment began, this declined from 0.15 in year + 1 to 0.02 in year + 6. Comparing corresponding years before and during LA treatment within a 12-year period (year − 6 to year + 6), all pairs exhibited a MACE reduction being essentially identical to the mean reduction calculated with all events for the entire observation period.

### Safety and tolerability of LA treatment

During 36,745 LA treatments, there were unexpected adverse events in 1.1 %, vascular problems in 2.1 %, and technical problems in 0.08 % of cases (for details, see [13]). Overall, 99.5 % of all treatments reached their treatment target, defined by the plasma or blood volume intended to treat.

### Termination of chronic LA treatment

LA treatment was terminated during the observation period for 27 of the total 118 patients. Reasons for termination are summarized in Table 6. Chronic LA treatment was halted in four patients at their request, resulting in treatment adherence rate of 99.5 % per year.

**Table 6:** Reason for terminating chronic lipoprotein apheresis (LA) in the cohort of 118 patients covering 797 patient years

Reason	Number of patients terminating LA	Relation to total number of patient years
Total	27	
Death	15	1 per 53 patient years
Dialysis patients	6	1 per 4 patient years
Non-dialysis patients	9	1 per 86 patient years
Cardiac death	7	1 per 114 patient years (8,8% per 10 patient years)
Progressive malignant disease	6	
Change of patient’s residence	2	
Patient’s will	4	1 per 200 years

## Discussion

In this retrospective study, 118 patients with established indication for lipoprotein apheresis due to HLP with high CVD risk were analyzed, who received more than 36,000 LA treatment sessions for an average time period of nearly 7 years. LA demonstrated to be an effective, safe, and well-tolerated extracorporeal treatment. Immediate effect of LA was lowering of both LDL-C and Lp(a) levels by an average of 67 %, irrespective of the initial level. Regarding time-averaged levels in the long-term, LA showed significantly less LDL-C reduction of 32 % in patients with severe hypercholesterolemia compared with 53 % long-term Lp(a) reduction in patients with isolated Lp(a)-HLP. The patients who started Lp(a) apheresis treatment with an already low LDL-C level of 96 mg/dL had an even faster LDL-C rebound and a correspondingly smaller long-term reduction rate of only 23 %. A similar observation of 18 % long-term LDL-C reduction was noted in the Pro(a)LiFe study [11]. In accord with Jaeger et al. [10], who reported a 36 % long-term Lp(a) reduction, but in contrast to our own findings (53 %), authors of this study also noted a lower long-term Lp(a) reduction of 32 %. In all three investigated populations, lowering of MACE rates after commencing extracorporeal Lp(a) elimination was equally impressive, i.e., by 78 % [11], by 86 % [10], and by 90 %, respectively.

Approximately 70 % of LA patients across Germany are treated due to severe hypercholesterolemia and 30 % due to isolated Lp(a)-HLP [15], which fully matches our patient population in proportion. The initial risk for our patients with high LDL-C levels was markedly lower (0.65) in the year prior to LA treatment than for Lp(a) patients (1.49). MACE reduction with LA treatment was lower in LDL-C patients (73.7 %) than in the Lp(a) group (90.4 %). This appears to confirm the conclusion that the higher the initial risk, the greater the effect of LA treatment. This relationship has already been observed between statin therapy and hypercholesterolemia leading to the number needed to treat. In the Pro(a)LiFe patient population, only three patients needed LA treatment for 1 year to avoid a MACE [11], which matches our findings.

In addition to the lipoprotein-eliminating effect of LA, a number of pleiotropic, especially pro-rheological and anti-inflammatory effects, must be mentioned [16]. It could be hypothesized that also the pulsatile fall in lipoproteins to extremely low values immediately after LA treatment might have a sustained positive effect on the vascular endothelium, resulting in a reduction of cardiovascular events more than double as high as expected from LDL-C elimination by statins alone [2, 17].

The clinical efficacy of LA can also be discussed in terms of patient mortality. Even if there are no mortality data available that can be compared with the patient population being investigated here, it becomes apparent that the cardiac mortality rate of just one death in 114 treatment years in a patient group with an initial average age of 60 years and a high risk for CVD is unexpectedly low.

Our study confirmed the safety of LA treatment, as it has been documented in more than 2,500,000 treatments worldwide. This is reflected by the total rate of only 3.3 % of unexpected adverse events, vascular access problems, and technical problems, and a completion rate of 99.5 % of all treatments.

## Conclusions

LA treatment of patients with a high risk for CVD due to LDL- and/or Lp(a)-HLP is effective, safe, and well tolerated. The number of cardiovascular events, at least during a 6-year period, was lowered by 80 %, which is a significant clinical benefit for these high-risk patients. Epidemiological research and prospective studies comparing LA and new Lp(a)-lowering medications might provide further insight into the role of Lp(a) in cardiovascular diseases.

### Conflict of interest

F. Heigl received lecture fees from B. Braun, Melsungen, Diamed, Cologne, Fresenius medical Care, Bad Homburg.

R. Klingel received financial support for clinical research activities by grants from Asahi Kasei Medical, Japan and Diamed, Germany.
